# Sex-Specific Clinical Characteristics and Long-Term Outcomes in Patients With Myocardial Infarction With Non-obstructive Coronary Arteries

**DOI:** 10.3389/fcvm.2021.670401

**Published:** 2021-06-09

**Authors:** Side Gao, Wenjian Ma, Sizhuang Huang, Xuze Lin, Mengyue Yu

**Affiliations:** Department of Cardiology, National Center for Cardiovascular Diseases, Fuwai Hospital, Chinese Academy of Medical Sciences and Peking Union Medical College, Beijing, China

**Keywords:** myocardial infarction with non-obstructive coronary arteries, sex difference, cardiovascular outcome, baseline characteristics, coronary artery disease

## Abstract

**Background:** Sex differences in clinical profiles and prognosis after acute myocardial infarction have been addressed for decades. However, the sex-based disparities among patients with myocardial infarction with non-obstructive coronary arteries (MINOCA) remain largely unreported. Here, we investigated sex-specific characteristics and long-term outcomes in MINOCA population.

**Methods:** A total of 1,179 MINOCA patients were enrolled, including 867 men and 312 women. The mean follow-up was 41.7 months. The primary endpoint was a composite of major adverse cardiovascular events (MACE), including all-cause death, non-fatal reinfarction, revascularization, non-fatal stroke, and hospitalization for unstable angina or heart failure. Baseline data and outcomes were compared. Kaplan-Meier curves and Cox regression analyses were used to identify association between sex and prognosis.

**Results:** Female patients with MINOCA had more risk profiles with regard to older age and higher prevalence of hypertension and diabetes compared with men. The evidence-based medical treatment was similar in men and women. The incidence of MACE (men vs. women: 13.8 vs. 15.3%, *p* = 0.504) did not differ significantly between the sexes. The Kaplan-Meier analysis also indicated that women had a similar incidence of MACE compared to men (log rank *p* = 0.385). After multivariate adjustment, female sex was not associated with the risk of MACE in overall (adjusted hazard ratio 1.02, 95% confidence interval: 0.72–1.44, *p* = 0.916) and in subgroups of MINOCA patients.

**Conclusion:** The long-term outcomes were similar for men and women presenting with MINOCA despite older age and more comorbidities in women. Future research should aim to improve in-hospital and post-discharge care for both sexes with MINOCA.

## Introduction

The sex differences in clinical presentation, treatment and outcomes of patients with acute myocardial infarction (AMI) have been investigated for decades (1–11). Generally, women with AMI have a greater burden of comorbidity and atypical symptoms than men. Women may also experience longer delays to reperfusion and are less likely to receive cardiac catheterization (1–11). Some studies report a higher unadjusted mortality for women after AMI, which is mainly explained by differences in age, comorbidities and use of guideline-based treatment (3–7). Meanwhile, others claim that the adjusted rates of mortality and cardiovascular (CV) events for men and women are similar, suggesting that both sexes can benefit from the current developed therapies (8–11). Even though these research enrolled a large sample of AMI, few studies have focused on the patients with myocardial infarction with non-obstructive coronary arteries (MINOCA).

As previously reported, MINOCA occurs in 5–10% of AMIs and disproportionately affects the younger and female in comparison to those with obstructive coronary artery disease (CAD) (12–15). The underlying reasons of MINOCA are varied and may include plaque rupture or erosion, thrombosis, spasm, embolization, dissection, microvascular dysfunction, and supply/demand mismatch. Other non-ischemic diseases such as acute myocarditis may also mimic the clinical presentation of MINOCA (13–15). Till now, the characteristics and long-term outcomes in Chinese population with MINOCA remain undetermined, and less is known about the sex disparities in this distinct entity. Here, we aimed to characterize the MINOCA population and find whether sex gaps in clinical profiles, management and prognosis exist for MINOCA in the contemporary practice.

## Methods

### Study Population

**Figure 1:**
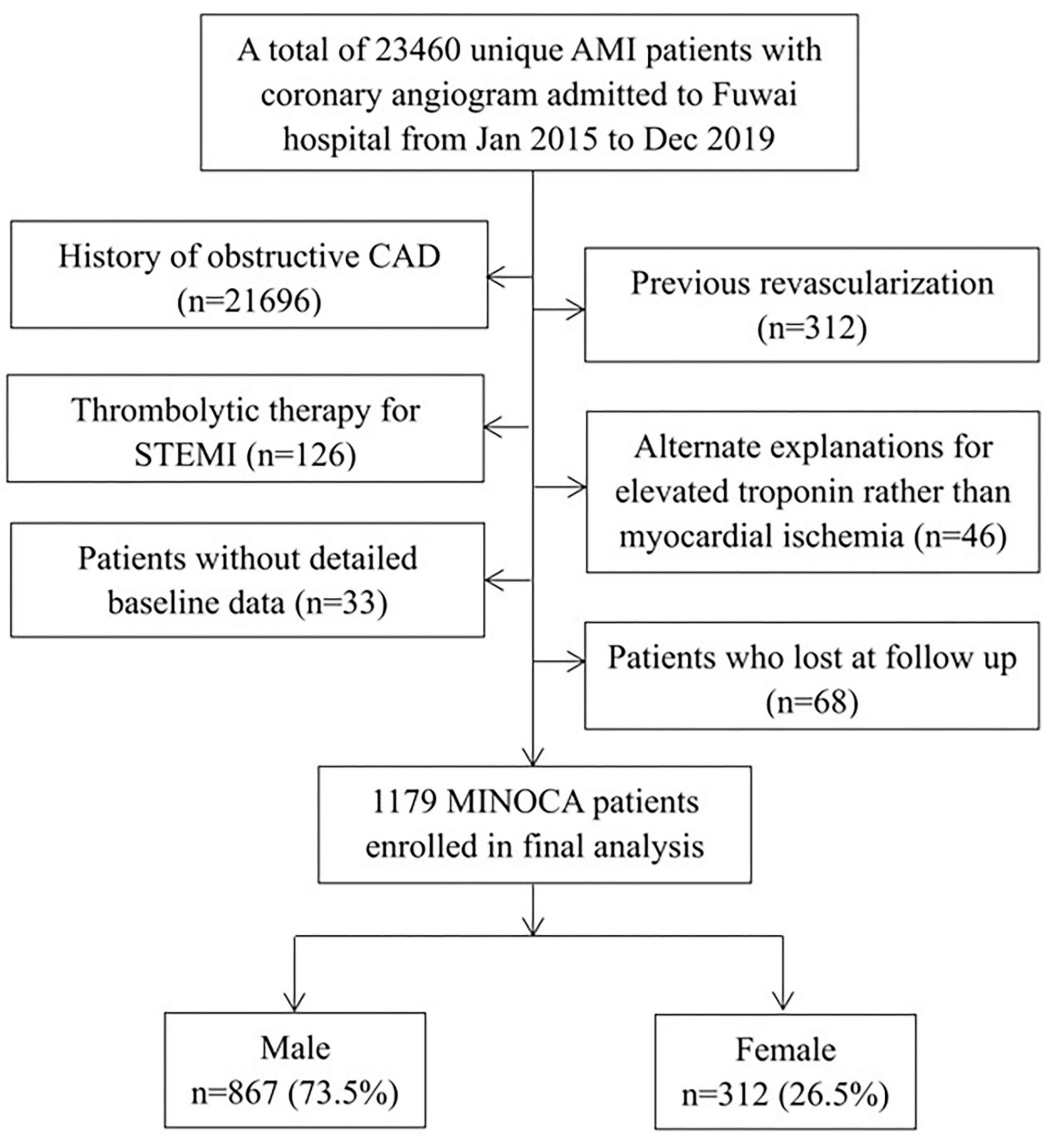
Study flowchart.

This was a single-center, prospective and observational study conducted in Fuwai Hospital and National Center for Cardiovascular Diseases. Patients were identified as having MINOCA if the confirmed diagnosis met the 4th universal definition of AMI (16) and the coronary angiogram performed during the index hospitalization did not show a stenosis of ≥50% in epicardial coronaries (13). Overall, 23,460 unique AMI patients with coronary angiography, including ST-segment elevation myocardial infarction (STEMI) and non-ST-segment elevation myocardial infarction (NSTEMI), were consecutively admitted to Fuwai hospital from Jan 2015 to Dec 2019. The following patients were excluded due to: (1) obstructive CAD (*n* = 21,696); (2) prior revascularization (*n* = 312); (3) thrombolytic therapy for STEMI since the degree of prior stenosis may change after thrombolysis (*n* = 126); (4) alternate reasons for elevated troponin rather than coronary-related ischemia (*n* = 46, e.g., heart failure, myocarditis, pulmonary embolism, takotsubo syndrome); (5) lack of detailed baseline data (*n* = 33); (6) lost at follow up (*n* = 68). As a result, a total of 1,179 MINOCA patients were enrolled in final analysis (Figure 1). Patients were treated with optimal medical therapies according to current guidelines, including dual anti-platelet therapy (DAPT), statins, β-blockers, and angiotensin-converting enzyme inhibitor (ACEI) or angiotensin receptor antagonist (ARB) (17, 18). This study was approved by Ethics Committee of Fuwai hospital and was conducted in accordance with the Declaration of Helsinki. All patients provided the written informed consent.

### Data Collection

Baseline characteristics regarding the demographic, clinical and laboratory data were obtained from in-person interviews and medical records. Body mass index (BMI) was calculated as weight (kg) divided by height (m) squared. Glycated hemoglobin (HbA_1c_) was tested with a liquid chromatography analyzer. Serum concentrations of creatinine, low density lipoprotein cholesterol (LDL-C), and high-sensitive C-reactive protein (hs-CRP) were measured with an automatic biochemistry analyzer. The N-terminal B-type natriuretic peptide (NT-proBNP) at admission and peak cardiac troponin I (TnI) values were recorded. The left ventricular ejection fraction (LVEF) was measured using the biplane Simpson method with echocardiography.

### Definitions and Outcomes

In the present study, diabetes (DM) was defined as having a history of DM or newly diagnosed DM with fasting blood glucose ≥ 7.0 mmol/L or 2-h plasma glucose ≥ 11.1 mmol/L (19). Hypertension was defined as repeated systolic blood pressure ≥ 140 mmHg or diastolic blood pressure ≥ 90 mmHg (at least two times in different environments) or currently taking anti-hypertensive drugs (20). Dyslipidemia was defined as LDL-C ≥ 3.4 mmol/L, high density lipoprotein cholesterol <1.0 mmol/L, triglyceride ≥ 1.7 mmol/L or patients who were taking lipid-lowering medication (21).

The primary endpoint of this study was major adverse cardiovascular events (MACE) defined as a composite of all-cause death, non-fatal MI, revascularization, non-fatal stroke, and hospitalization for unstable angina (UA) or heart failure (HF). The MACE was evaluated as time to first event. The secondary endpoints included each component of MACE and the composite “hard” endpoint of death, non-fatal MI, revascularization, or stroke. Of these, reinfarction was diagnosed based on the 4th universal definition of MI (16). Revascularization, mainly referred to percutaneous coronary intervention (PCI), was performed at the operator's discretion due to recurrent ischemia and progression of coronary stenosis. Stroke was defined by the presence of typical symptoms and imaging (22). Hospitalization for UA or HF represented the clinical status and quality of life after AMI. Specifically, UA was diagnosed if the symptoms exacerbated with an increase in severity or length of anginal attacks (18). HF was defined with the typical symptoms and evidence of a structural or functional cardiac abnormality (22). Patients were regularly followed up at clinics or through telephone by well-trained cardiologists or nurses who were blinded to the purpose of this study. The endpoints were checked and confirmed by at least two professional physicians.

### Statistical Analysis

The data were expressed as mean ± standard deviation or median with interquartile range for continuous variables and the number with percentage for categorical variables. Differences were assessed using Student's *t*-test or Mann-Whitney U test for continuous variables and Pearson's χ^2^ or Fisher's exact test for categorical variables. The survival curves indicating cumulative incidence of events among male and female groups were conducted using the Kaplan-Meier analysis and compared by log-rank test. Univariate and multivariate Cox proportional regression analyses were used to identify association between sex and event risk. Clinically relevant and prognosis-related variables among groups were enrolled into the multivariate model, including age, MI type (NSTEMI or STEMI), presence of hypertension, diabetes and dyslipidemia. The hazard ratio (HR) with 95% confidence interval (CI) were calculated. At subgroup analysis, patients were further stratified according to age, BMI, MI type, hypertension, diabetes, dyslipidemia, and LVEF level, and the female-to-male unadjusted risk of MACE was calculated. All tests were 2-tailed, and *P* < 0.05 was considered significant. The statistical analyses were performed using SPSS V.22.0 (SPSS Inc., Chicago, Illinois, USA).

## Results

### Baseline Characteristics

Patients with MINOCA were stratified by their sex, including 867 men and 312 women (Figure 1). As shown in Table 1, female patients tended to be older and non-smoker. They were less likely to present with STEMI and had more prevalence of hypertension and diabetes. Women with MINOCA also had lower BMI, higher HbA_1c_ and lower peak TnI values compared with men. There were no significant differences in vital signs at admission, Killip class, LVEF level, serum creatinine, LDL-C, hs-CRP, and NT-proBNP values between the sexes. Both men and women have similar rates to receive emergent angiography and the evidence-based medication at discharge.

**Table 1:** Baseline characteristics among male and female MINOCA patients.

**Variable**	**All MINOCA (*n* = 1,179)**	**Male (*n* = 867)**	**Female (*n* = 312)**	***P*-value**
Age, years	55.7 ± 11.8	54.1 ± 11.7	58.8 ± 10.3	<0.001
BMI, kg/m^2^	25.4 ± 3.7	25.7 ± 3.4	24.7 ± 4.4	<0.001
STEMI, *n* (%)	475 (40.2%)	379 (43.7%)	96 (30.7%)	<0.001
Emergent angiography, *n* (%)	159 (13.5%)	107 (12.3%)	52 (16.6%)	0.069
**Vital signs at admission**				
Systolic BP, mmHg	125.3 ± 17.5	124.8 ± 17.0	126.7 ± 18.8	0.095
Diastolic BP, mmHg	76.7 ± 11.7	77.0 ± 11.7	76.2 ± 11.5	0.103
Heart rate, bpm	69.5 ± 10.9	69.2 ± 10.8	70.1 ± 11.2	0.206
**Medical history**, ***n*** **(%)**				
Hypertension	630 (53.4%)	439 (50.6%)	191 (61.2%)	0.001
Diabetes	187 (15.9%)	123 (14.1%)	64 (20.5%)	0.009
Dyslipidemia	686 (58.2%)	497 (57.3%)	189 (60.5%)	0.318
Previous MI	58 (4.9%)	48 (5.5%)	10 (3.2%)	0.103
Smoking	483 (40.9%)	462 (53.2%)	21 (6.7%)	<0.001
LVEF (%)	60.5 ± 7.5	60.3 ± 7.8	60.9 ± 6.4	0.245
Killip class ≥ 2, *n* (%)	89 (7.5%)	65 (7.4%)	24 (7.6%)	0.834
**Laboratory data**				
HbA_1c_, %	5.98 ± 0.98	5.94 ± 0.99	6.09 ± 0.95	0.019
LDL-C, mmol/L	2.29 ± 0.76	2.27 ± 0.77	2.34 ± 0.73	0.138
Creatinine, μmol/L	83.13 ± 15.89	82.62 ± 13.82	84.36 ± 17.28	0.301
hs-CRP, mg/L	2.20 (1.03, 5.75)	2.14 (1.06, 5.46)	2.38 (0.92, 6.51)	0.290
NT-proBNP, pg/mL	372 (112, 683)	369 (109, 673)	376 (115, 688)	0.115
Peak TnI, ng/mL	3.24 (0.72, 6.51)	3.37 (0.81, 6.98)	3.13 (0.65, 5.94)	0.032
**Medication at discharge**, ***n*** **(%)**				
DAPT	1,091 (92.5%)	809 (93.3%)	282 (90.3%)	0.108
Statin	1,130 (95.8%)	831 (95.8%)	299 (95.8%)	0.693
Beta-blocker	860 (72.9%)	632 (72.8%)	228 (73.0%)	0.951
ACEI or ARB	759 (64.4%)	571 (65.8%)	188 (60.2%)	0.076

*BMI, body mass index; STEMI, ST-segment elevation myocardial infarction; BP, blood pressure; LVEF, left ventricular ejection fraction; HbA_1c_, glycated hemoglobin; LDL-C, low density lipoprotein-cholesterol; hs-CRP, high-sensitive C-reactive protein; NT-proBNP, N-terminal B-type natriuretic peptide; TnI, Troponin I; DAPT, dual anti-platelet therapy; ACEI, angiotensin-converting enzyme inhibitor; ARB, angiotensin receptor antagonist*.

### Clinical Outcomes

During the mean follow-up time of 41.7 months, 168 MINOCA patients experienced MACE (18 died, 41 had recurrent MI, 46 had revascularization, 12 suffered stroke, 71 was hospitalized for UA and 48 hospitalized for HF) (Table 2). Female patients had a similar incidence of MACE compared with male (men vs. women: 13.8 vs. 15.3%, *p* = 0.504). The incidence of the composite hard endpoint of death, MI, revascularization or stroke (men vs. women: 8.3 vs. 9.6%, *p* = 0.480) and the other component event (all *p* > 0.05) also did not differ significantly between the two groups.

**Table 2:** Clinical outcomes among male and female MINOCA patients.

**Variable**	**All MINOCA (*n* = 1,179)**	**Male (*n* = 867)**	**Female (*n* = 312)**	***P*-value**
Mean follow-up time, months	41.7 ± 18.4	42.1 ± 18.3	41.4 ± 18.6	0.126
**Clinical outcomes**, ***n*** **(%)**				
MACE	168 (14.2%)	120 (13.8%)	48 (15.3%)	0.504
Death, non-fatal MI, non-fatal stroke or revascularization	102 (8.6%)	72 (8.3%)	30 (9.6%)	0.480
All-cause death	18 (1.5%)	13 (1.4%)	5 (1.6%)	0.899
Non-fatal MI	41 (3.4%)	28 (3.2%)	13 (4.1%)	0.438
Revascularization	46 (3.9%)	30 (3.4%)	16 (5.1%)	0.192
Non-fatal stroke	12 (1.0%)	9 (1.0%)	3 (0.9%)	0.908
Hospitalization for UA	71 (6.0%)	51 (5.8%)	20 (6.4%)	0.737
Hospitalization for HF	48 (4.0%)	36 (4.1%)	12 (3.8%)	0.815

*MACE, major adverse cardiovascular events; MI, myocardial infarction; UA, unstable angina; HF, heart failure*.

**Figure 2:**
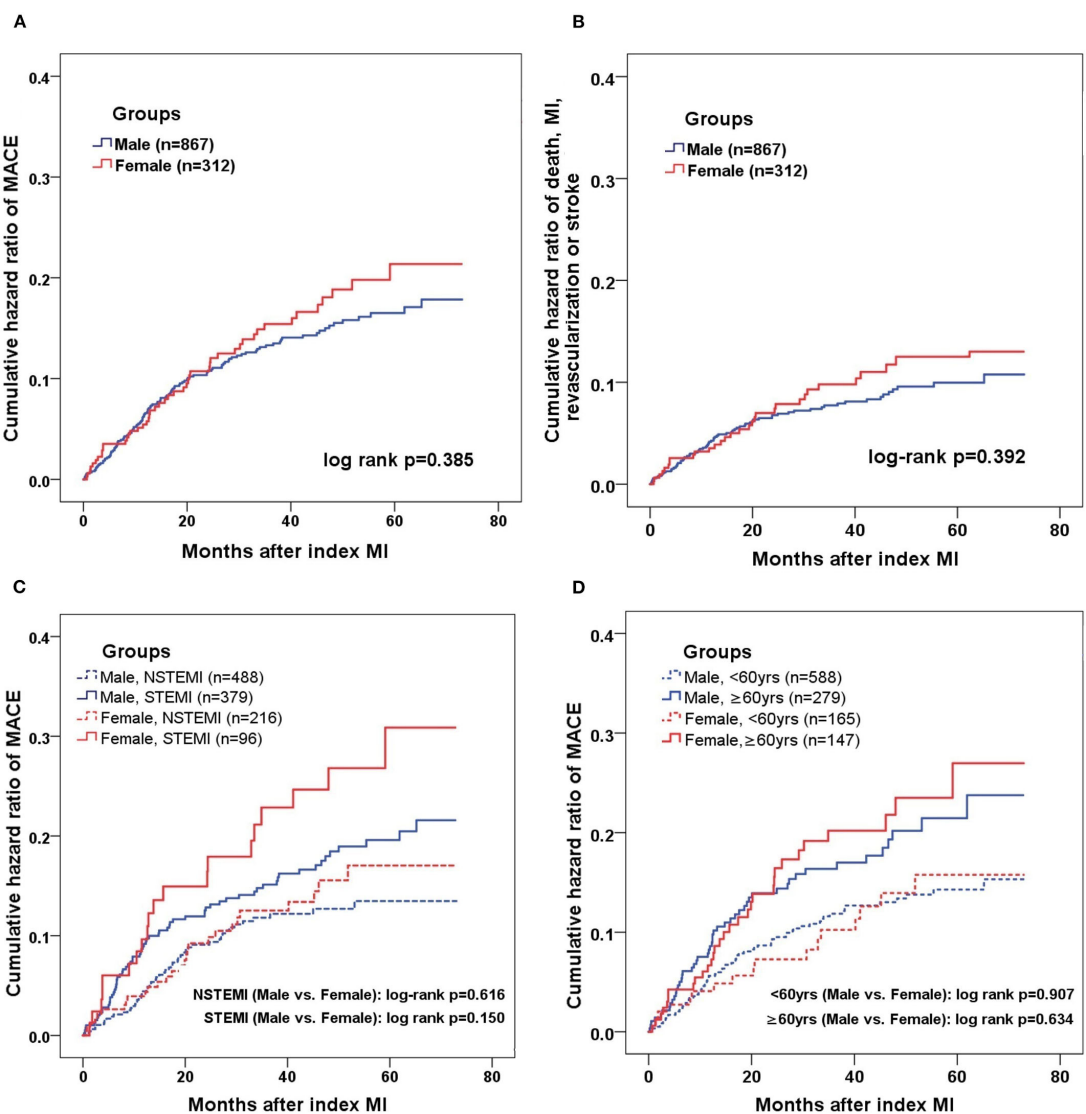
Kaplan-Meier analysis of MACE in male and female MINOCA patients. Kaplan-Meier curves showing the cumulative incidence of MACE **(A)** and composite “hard” endpoint of death, non-fatal MI, non-fatal stroke, or revascularization **(B)** in men and women presenting with MINOCA. Male and females were further stratified by MI type **(C)** and age **(D)**. The threshold of 60 years was used to define the younger or older. MACE included all-cause death, non-fatal MI, revascularization, non-fatal stroke, and hospitalization for unstable angina or heart failure. STEMI, ST-segment elevation myocardial infarction; NSTEMI, non-ST-segment elevation myocardial infarction.

The Kaplan-Meier curves showed that female had slightly higher rates of MACE and the composite hard endpoint than men, however, the discrepancies were not significant (log rank *p* = 0.385 and 0.392, respectively) (Figures 2A,B). The male and female patients were further stratified by the age (<60 or ≥60 years) and MI type (NSTEMI or STEMI). Whereas, patients with STEMI and older age had more cumulative incidence of MACE, the prognostic difference between the sexes remained non-significant in each subgroup (men vs. women: log rank *p* = 0.616 and 0.150 for NSTEMI and STEMI; log rank *p* = 0.907 and 0.634 for the younger and older group) (Figures 2C,D).

### Association Between Gender and Outcomes

**Figure 3:**
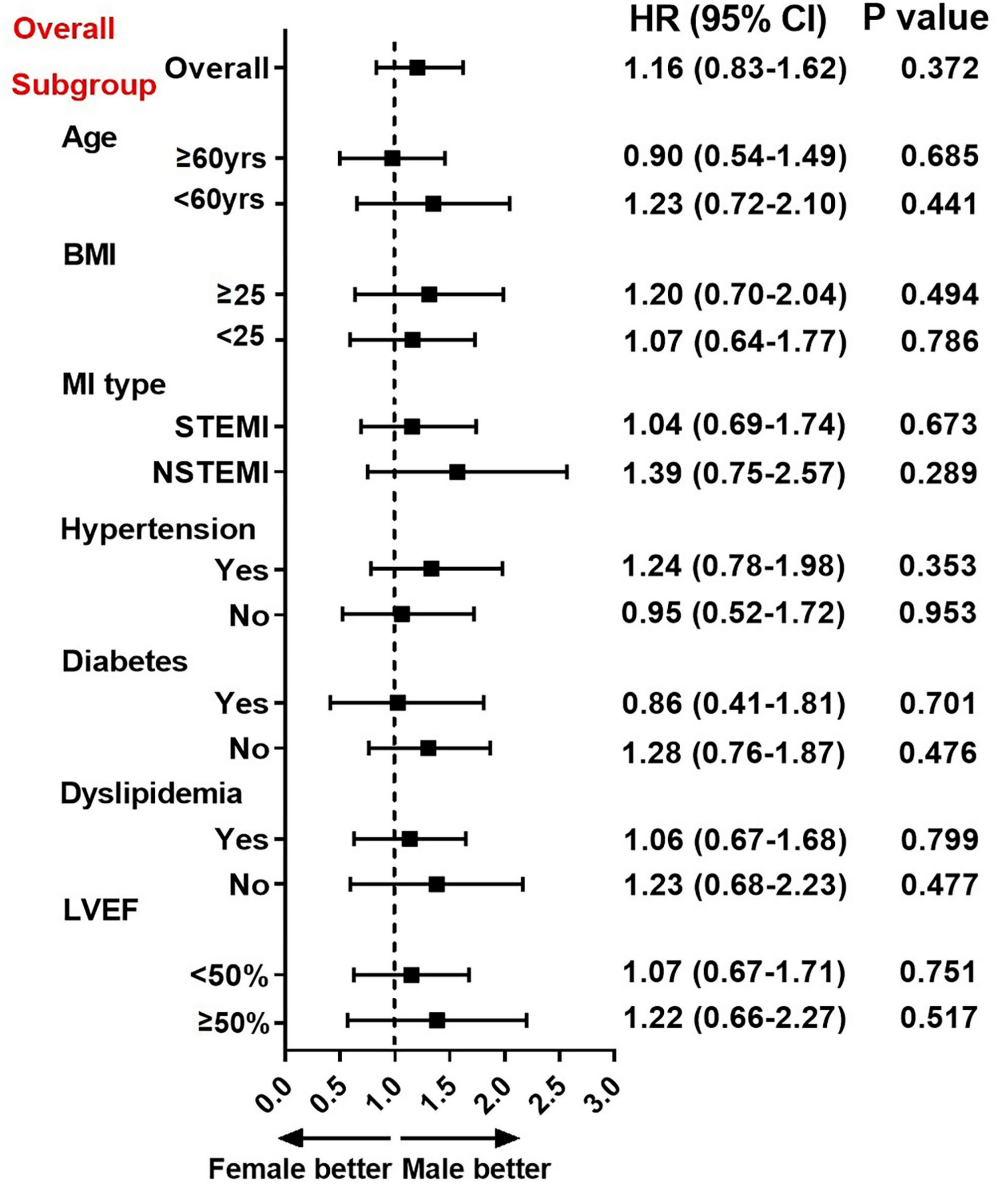
Association between gender and risk of MACE in overall and subgroups. Subgroup analysis showing the association between sex and risk of MACE in subsets of MINOCA patients. Hazard ratio (HR) was expressed as female-to-male risk ratio and calculated by univariate Cox regression analysis. The vertical dotted line indicated the HR value of 1. CI, confidence interval; BMI, body mass index; LVEF, left ventricular ejection fraction; STEMI, ST-segment elevation myocardial infarction; NSTEMI, non-ST-segment elevation myocardial infarction.

At Cox regression analysis (Table 3), there were no significant differences either in unadjusted or age-adjusted risk of events (all *p* > 0.05) between sexes. After multivariate adjustment, female sex was not associated with the risk of MACE (HR = 1.02, 95% CI: 0.72–1.44, *p* = 0.916) and the composite endpoint of death, MI, revascularization or stroke (HR = 0.94, 95% CI: 0.60–1.47, *p* = 0.788). Further, the risk of MACE remained similar for men and women in a variety of subsets of MINOCA stratified by the age, BMI, MI type, hypertension, diabetes, dyslipidemia, and LVEF level (all *p* > 0.05) (Figure 3).

**Table 3:** Association between gender and outcomes in MINOCA.

**Cox analysis model**	**MACE**		**Death, non-fatal MI, non-fatal stroke or revascularization**	
	**HR (95% CI)**	***P*-value**	**HR (95% CI)**	***P*-value**
Unadjusted	1.16 (0.83–1.62)	0.372	1.20 (0.78–1.84)	0.393
Age adjustment	1.06 (0.74–1.50)	0.743	0.97 (0.63–1.51)	0.924
Multivariate adjustment	1.02 (0.72–1.44)	0.916	0.94 (0.60–1.47)	0.788

*MACE, major adverse cardiovascular events; MI, myocardial infarction; HR, hazard ratio; CI, confidence interval. HR was calculated as female compared with male. HR was adjusted for age, MI type (NSTEMI or STEMI), presence of hypertension, diabetes, and dyslipidemia in the multivariate model*.

## Discussion

In the present study, we described the sex-based clinical characteristics in MINOCA subpopulation and found that the long-term outcomes were similar for men and women presenting with MINOCA. Our data highlight the opportunities to improve healthcare for both sexes in this cohort who remain at considerable CV risks in the contemporary real-world management of MINOCA.

It is increasingly recognized that a proportion of patients with AMI actually have no significant obstructive coronary artery lesions, and the term MINOCA has been coined to describe this distinct entity (13). The underlying mechanisms of MINOCA are multiple, including coronary (plaque rupture, spasm, etc.) and non-coronary causes (myocarditis, etc.). More recently, MINOCA has been primarily used to describe those with coronary-related ischemia (14). We adopted this criteria and established a MINOCA cohort with long-term follow-up. The prevalence of MINOCA in our study was nearly 5.1% among AMIs, which is close to the estimated prevalence of 5–10% (13). As reported, about one-third of MINOCA were classified as STEMI (15). Compared with those with obstructive AMI, patients with MINOCA were more likely to be younger (median age of 55 years), female (rate of 40%) and had fewer comorbidities (15). In the present study, we described the baseline characteristics of MINOCA as well (female of 26.5%, mean age of 55.7 years, STEMI of 40.2%, hypertension of 53.4%, diabetes of 15.9%, dyslipidemia of 58.2%). These data were generally consistent with previous literature, which may help us better understand the clinical profiles of MINOCA. Yet, the ratio of women was relatively low in our study, partially due to the large proportion of men in overall AMIs treated in our center and a lower rate for women to receive coronary angiography. Still, we identified hundreds of women presenting with MINOCA and they deserve to be well-described. Future nationwide registry cohorts of MINOCA are also needed to validate our findings.

It may seem reasonable for MINOCA patients to have a better prognosis than those with MI and significant CAD (23, 24), however, the rate of adverse CV events is not trivial in MINOCA, especially considering that they are younger and have fewer baseline risk factors. Recent studies confirmed that patients with MINOCA were still at considerable risk for 1-year mortality and the occurrence of MACE (23–28). In line with these findings, we found that 1.5% of MINOCA patients died and 14.2% of them experienced MACE over the mean follow-up of 3.5 years. These data highlight the challenge for physicians to improve the provision of healthcare for this population.

Over the past decade, studies have reported conflicting results on sex differences in clinical profiles and outcomes in patients with AMI. Generally, women are reported to be older, have more comorbidities and have lower rates of receiving PCI and evidence-based treatment than men (2–11). The unadjusted in-hospital and long-term mortality in women tend to be higher than in men, and this difference is more pronounced in STEMI subpopulation (3–7). However, this sex disparity in mortality became attenuated or even non-significant after age or multivariate adjustment (8–11). Recently, several nationwide trend analyses also revealed that there was no difference in adjusted mortality in women compared to men although women had more comorbidities (9, 10). Despite these results and ongoing studies, few of them have focused on patients with MINOCA and explored potential sex gaps in clinical characteristics and outcomes in this specific population.

Our study addressed this issue and consistently, we found that women with MINOCA were older, had lower presence of STEMI, and had more prevalence of hypertension and diabetes compared with men. Further, we did not observe significant sex disparities in crude and adjusted prognosis after MINOCA, which was in line with previous studies. A national registry found that no sex difference in mortality was observed in MINOCA (29). Another study also proved that women and men diagnosed with MINOCA revealed similar in-hospital outcomes (30). There might be possible reasons for this result. First, many risk factors were comparable among men and women. In our cohort, both sexes had similar clinical conditions such as vital signs at admission, Killip class, and cardiac functions (e.g., LVEF). Second, the rates of receiving secondary prevention treatments were similar for men and women in our center. Accordingly, they obtained similar long-term beneficial effects of medical therapies. Third, given that the overall prognosis after AMI has been markedly improved with the advances in care for AMI, it is not surprising that the sex gaps in long-term outcomes have also reduced over time (31). Still, we found that among MINOCA subgroups, the elder women with STEMI seemed to have poorer outcomes. Special attention should be given to this subpopulation.

In clinical practice, MINOCA is not uncommon and represents a distinct entity of all AMIs. The sex-related outcome differences in this population may be less related to the sex itself but instead should be largely explained by differences in age, comorbidities, and treatment utilization. These factors may serve as effect modifiers and further affect prognosis among men and women differently. In this regard, we should recognize them at early stages and take pre-emptive measures in order to find opportunities to improve care in women, especially those at high risks. On one hand, physicians should consider MINOCA as a heterogeneous working diagnosis that requires further evaluation with multi-modality imaging to find the underlying causes and thus tailor targeted treatment. On the other hand, the use of evidence-based treatment in women should be emphasized, and there is an enduring need to reduce or even eliminate the sex disparities in quality of care.

## Limitation

Several limitations should be acknowledged. First, we enrolled more than a thousand subjects and this sample size was relatively considerable for MINOCA. Even through, the present data were derived from a single center, and our findings need to be verified by multicenter and larger cohort studies. Second, given the nature of sex, our analysis revealed critical associations but could not prove causation. Third, despite multivariate adjustment and subgroup analyses were performed, there might be other measured or unmeasured confounding variables that would have modified the relationship between sex and prognosis. Fourth, we did not capture the exact mechanism for every MINOCA patient. Future research are warranted to identify the etiology of MINOCA and find the sex-related outcomes in different phenotypes of MINOCA. Further, we did not record the percentage of menopausal women in all patients. The relation of menopause status with CV outcomes in MINOCA should also be addressed by future studies.

## Conclusion

Female patients with MINOCA tended to be older, less likely to present with STEMI, and had more prevalence of hypertension and diabetes compared with men. Both sexes received a similar rate of evidence-based medication. After multivariate adjustment, the female sex was not associated with the long-term risk of MACE after MINOCA. Future nationwide quality control programs are warranted to discover and possibly narrow the sex-related disparities in quality of care and outcomes in MINOCA population.

## Data Availability Statement

The original contributions presented in the study are included in the article/supplementary material, further inquiries can be directed to the corresponding author/s.

## Ethics Statement

The studies involving human participants were reviewed and approved by Ethics Committee of Fuwai hospital. The patients/participants provided their written informed consent to participate in this study.

## Author Contributions

SG conceived and designed the study and drafted the manuscript. SG, WM, SH, and XL performed data analysis and interpretation. MY reviewed and gave final approval of the version to be published. All authors read and approved the final manuscript.

## Conflict of Interest

The authors declare that the research was conducted in the absence of any commercial or financial relationships that could be construed as a potential conflict of interest.
